# Metabolomics-based discovery of XHP as a CYP3A4 inhibitor against pancreatic cancer

**DOI:** 10.3389/fphar.2023.1164827

**Published:** 2023-04-04

**Authors:** Yuting Yang, Yanlei Guo, Hua Luo, Menglei Wang, Fang Chen, Huawei Cui, Ping Chen, Zhujun Yin, Li Li, Ying Dai, Jin Zeng, Junning Zhao

**Affiliations:** ^1^ College Pharmacy, Chengdu University of Traditional Chinese Medicine, Chengdu, Sichuan, China; ^2^ Translational Chinese Medicine Key Laboratory of Sichuan Province, Sichuan Academy of Chinese Medicine Sciences, Sichuan Institute for Translational Chinese Medicine, Chengdu, Sichuan, China; ^3^ Macau Centre for Research and Development in Chinese Medicine, State Key Laboratory of Quality Research in Chinese Medicine, Institute of Chinese Medical Sciences, University of Macau, Macao, China

**Keywords:** pancreatic cancer, Xihuang pills, CYP3A4, widely targeted metabolomics, steroid hormone biosynthesis

## Abstract

**Background:** Xihuang Wan (XHW), a purgative and detoxifying agent, is commonly utilized in modern medicine as a treatment and adjuvant therapy for various malignancies, including breast cancer, liver cancer, and lung cancer. A clinical study demonstrated the potential usefulness of the combination of XHW and gemcitabine as a therapy for pancreatic cancer (PC), indicating that XHW’s broad-spectrum antitumor herbal combination could be beneficial in the treatment of PC. However, the precise therapeutic efficacy of XHW in treating pancreatic cancer remains uncertain.

**Aim:** This study assessed the biological activity of XHW by optimizing the therapeutic concentration of XHW (Xihuang pills, XHP). We performed cell culture and developed an animal test model to determine whether XHP can inhibit pancreatic cancer (PC). We also applied the well-known widely targeted metabolomics analysis and conducted specific experiments to assess the feasibility of our method in PC therapy.

**Materials and Methods:** We used UPLC/Q-TOF-MS to test XHP values to set up therapeutic concentrations for the *in vivo* test model. SW1990 pancreatic cancer cells were cultured to check the effect the anti-cancer effects of XHP by general *in vitro* cell analyses including CCK-8, Hoechst 33258, and flow cytometry. To develop the animal model, a solid tumor was subcutaneously formed on a mouse model of PC and assessed by immunohistochemistry and TUNEL apoptosis assay. We also applied the widely targeted metabolomics method following Western blot and RT-PCR to evaluate multiple metabolites to check the therapeutic effect of XHP in our cancer test model.

**Results:** Quantified analysis from UPLC/Q-TOF-MS showed the presence of the following components of XHP: 11-carbonyl-β-acetyl-boswellic acid (AKBA), 11-carbonyl-β-boswellic acid (KBA), 4-methylene-2,8,8-trimethyl-2-vinyl-bicyclo [5.2.0]nonane, and (1S-endo)-2-methyl-3-methylene-2-(4-methyl-3-3-pentenyl)-bicyclo [2.2.1heptane]. The results of the cell culture experiments demonstrated that XHP suppressed the growth of SW1990 PC cells by enhancing apoptosis. The results of the animal model tests also indicated the suppression effect of XHP on tumor growth. Furthermore, the result of the widely targeted metabolomics analysis showed that the steroid hormone biosynthesis metabolic pathway was a critical factor in the anti-PC effect of XHP in the animal model. Moreover, Western blot and RT-PCR analyses revealed XHP downregulated CYP3A4 expression as an applicable targeted therapeutic approach.

**Conclusion:** The results of this study demonstrated the potential of XHP in therapeutic applications in PC. Moreover, the widely targeted metabolomics method revealed CYP3A4 is a potential therapeutic target of XHP in PC control. These findings provide a high level of confidence that XHP significantly acts as a CYP3A4 inhibitor in anti-cancer therapeutic applications.

## 1 Introduction

Pancreatic cancer (PC) is one of the most significant neoplasms of the gastrointestinal tract. The prevalence of risk factors, including those related to stressful environments and the consumption of processed foods has been increasing dramatically (Rahib et al., 2014; Chen et al., 2016; Moore and Donahue, 2019). Worldwide, PC ranks first among cancer-related diseases and fourth and sixth in China (Rahib et al., 2021). Despite treatments and delivery methods such as chemotherapy, the survival rate of patients with PC is relatively low, with <10% of patients surviving for 5 years or more (He et al., 2022). Moreover, increasing the quality of life of patients with PC remains challenging.

Increasing attention has been paid to the use of herbal medicine such as traditional Chinese medicine in the treatment of various diseases including cancer. The advantages of herbal medicine-based therapy include multi-targeting, multi-channeling, structural stability, high safety, and minimal side effects (Rawla et al., 2019; Wang et al., 2021). Herbs such as emodin, matridin, or triptolide showed significant improvement in therapeutic applications and reduced the adverse effects of radiotherapy in the treatment of PC (Lang et al., 2020; Wei et al., 2011; Chen et al., 2013; Liu et al., 2014). Moreover, f QYHJ and Fufangkushen are among those herbs used in the successful therapy for PC (Liu et al., 2003).

Excessive dampness, heat, and toxins together with unknown factors may contribute to serious diseases. (Jin and Wang., 2014). For instance, the aforementioned factors are risk factors for PC formation; however, more research is needed to verify their effects. The present study tested the hypothesis that the use of Chinese herbal medicine for the treatment of PC clears heat, removes toxins, resolves dampness, and disperses accumulation. We herein introduce a detoxifying agent, XHW, which comprises Niuhuang (*Bos taurus* domesticus Gmelin, artificial Niuhuang in Chinese), Shexiang (*Moschus berezovskii* Flerov, *Moschus sifanicus* Przewalski, *Moschus moschiferus* Linnaeus, artificial Shexiang in Chinese), Ruxiang (*Boszvellia carterii* Birdw., *Boswcllia bhaurdajiana* Birdw., Ruxiang in Chinese), and Moyao (*Commiphora myrrha* Engl., *Commiphora molmol* Engl., Moyao in Chinese). XHW is primarily utilized for the treatment of various malignant tumors, including breast cancer (Xu et al., 2020; Wu and Wang., 2020), liver cancer (Zhou, Y., 2020; Liu et al., 2010), and lung cancer (He et al., 2020; Chen, 2020; Cen, 2017) Combination treatment is among the significant therapeutic approaches in PC. A previous study comparing chemotherapy alone and the combination of XHW + chemotherapy showed that the combination therapy showed significant tumor inhibition and suppression of CA19-9, a PC biomarker. Reduced chemotherapy-induced leukopenia was also observed (Zhang et al., 2010).

The drawbacks of Chinese herbs include their complexity in formulation, which limits their wide applications in targeted therapy. However, recent technological developments in metabolomics, proteomics, genomics, and transcriptomics have provided new insights into the potential use of herbal medicines for disease treatment (Guo et al., 2022). Therefore, interest is increasing regarding the therapeutic applications of traditional Chinese medicine (Zhang et al., 2022; Wu et al., 2022; Pan et al., 2022).

In the present study, we formulated XHP, a novel agent derived from XHW, and assessed its effects on the treatment of PC in an *in vitro* cell culture and animal test model. We also followed up these findings by applying widely targeted metabolomics screening for validation of our target therapy together with Western blot and RT-PCR analyses. Our results revealed that XHP inhibited CYP3A4, downregulating its expression level and regulating related metabolic pathways in the animal model. Our findings demonstrate CYP3A4 as a significant therapeutic target and the clinical applications of Chinese herbal medicines and their compounds.

## 2 Materials and methods

### 2.1 Animals and cells

Six-week-old male BALB/c nude mice (weight 20 ± 2 g) were obtained from Beijing HFK Bioscience (Certificate of Conformity No. SYXK (chuan) 2008-100; China). Human SW1990 PC cells (Fuheng Biology, China) were cultured in L-15 medium (L20256, Fuheng Biology, China) supplemented with 10% fetal bovine serum (AUS-01S-02, CellBox, Australia) and 1% penicillin–streptomycin (15140122, Gibco, United States), and maintained in an incubator at 37°C, 95% O_2_–5% CO_2_. All experimental procedures were carried out in accordance with the ethical principles for laboratory animals (R20220210-2) as stipulated by the Science and Technology Department of Sichuan Province (Chengdu, China).

### 2.2 Drug preparation

XHP was supplied as samples from the Department of Pharmacy (Sichuan Academy of Chinese Medicine Sciences). For mass analysis, one XHP tablet was placed in a 10-mL volumetric flask and sonicated with methanol–acetonitrile–water (4:4:2) for 30 min and filtered using a 0.22-μm microporous filter membrane. For *in vivo* animal studies, an appropriate amount of XHP was crushed in a mortar and placed in a plastic bottle in CMC-Na 0.5% (CMC-Na: hot water = 1:200) supplied by KESHI (9004-32-4, China). The positive drug was gemcitabine hydrochloride for injection (CTTQ Pharmaceutical, China), with saline used as a solvent. For the cell assay, 3 g of XHP was placed in a 15 mL centrifuge tube and dissolved in 12 mL of DMSO. After filtration, a master batch of 250 mg/mL was obtained. The mother liquor was filtered through a filter membrane (0.22 μm), diluted to the appropriate dose, and stored at 4°C before use.

### 2.3 Chemicals and reagents

Cell Counting Kit-8 (CCK-8) was purchased from Biosharp (22082317, China), Hoechst 33258 from Beyotime (C1011, China), annexin-V FITC Apoptosis Kit from Beyotime (C1062L, China), antigen Ki67 from Servicebio (GB121141, China), 10% goat serum from Dr. D. Bio (AR1009, China), secondary antibody (HRP-labeled goat anti-rabbit, GB23303) and DAB kit from Beijing Zhongshan JinQiao Biological Co. Ltd. (ZLI-9018, China), BCA Kit from Beyotime (P0009, China), antibody CYP3A4 (rabbit) from CST (13384S, United States), GAPDH (mouse) and biotinylated goat anti-mouse IgG (H + L) from ABclonal (AC033; AS033, United States), biotinylated goat anti-rabbit IgG (H + L) from Affinity Biosciences (S0001, United States), Molpure® Cell/Tissue Total RNA Kit from YEASEN (19221ES50, China), PrimeScript RT reagent kit from Takara (RR047A, China), and TB Green TM Premix Ex Taq^TM^ Ⅱ (Tli RNaseH Plus) from Takara (RR820A, China).

### 2.4 UPLC/Q-TOF-MS for the qualitative detection of XHP components

We used a column system on an LC30A-UPLC device at the Shimadzu Laboratory of Japan, which utilized a Kinetex XB-C18 column (100 mm × 2.1 mm, 2.6 μm) with a mobile phase consisting of ultrapure water (A)—acetonitrile (B), containing 0.1% formic acid The gradient elution program started with 10% B for 1.00 min, followed by a change to 55% B from 1.00 to 7.00 min, and then to 85% B from 7.00 to 18.00 min, continuing until 22.00 min. Subsequently, the system was adjusted to 10% B from 22.5 min and continued until 25.00 min. The flow rate was set at 250 μL-min^-1^, and the column temperature was maintained at 30°C.

The Triple TOF 4600 Q-TOF-MS (AB SCIEX, United States) instrument uses an ESI ion source with two types of ionization that allow for both positive and negative ionization. The mass scan range of the instrument was set between 100 and 1,000 m/z with the following gas pressure settings: sheath gas, 0.38 MPa; auxiliary gas, 0.38 MPa; and curtain gas, 0.17 MPa. Atomization was achieved using TOF/MS primary pre-scan and the triggered secondary product Ion-IDA ion accumulation time, which were set to 250 and 100 ms, respectively. Multi-mass-deficit (MMDF) and dynamic background subtraction (DBS) were utilized as secondary trigger conditions. The declustering voltage was set to 80 V, and the collision energy (CE) was ±35 eV. The CES collision energies in the positive and negative modes were 35 ± 15 eV and −25 ± 15 eV, respectively. We determined the components of the XHP used in our experiment based on reference materials, such as 11-carbonyl-β-acetyl boswellic acid (AKBA), 11-carbonyl-β-boswellic acid (KBA), 4-methylene-2,8,8 -trimethyl-2-vinyl-bicyclo [5.2.0]nonane, and (1S-endo)-2-methyl-3-methylene-2-(4-methyl-3- pentenyl)-bicyclo [2.2.1heptane, among others (Yang et al., 2022). The XIC Manager function in Peak View 1.2 (AB SCIEX, United States) was used for preliminary screening of the compounds to gather information on molecular ion peaks, secondary fragmentation ions, and retention times. The compounds were then identified based on their accurate molecular masses in conjunction with a secondary spectral fragmentation analysis.

### 2.5 Cell inhibition rates

We performed a CCK-8 assay to determine the inhibition by XHP of SW1990 PC cells. The cells were cultured in 96-well plates (*n* = 6) at a density of 10^4^ cells/well and incubated at 37°C in a 95% O_2_, 5% CO_2_ incubator for 24 h. A blank control group was included and cells were treated with XHP solution at gradient concentrations of 74.5 μg/mL, 92.7 μg/mL, 116.36 μg/mL, 145.45 μg/mL, and 181.82 μg/mL. The cells were cultured for an additional 24, 48, and 72 h before performing the CCK-8 assays. Spectroscopy analysis was conducted to measure the optical density (OD) of the cells at 450 nm using an RC/STEEVOLYZER enzyme marker (Tecan Sunrise, Switzerland).

### 2.6 Hoechst 33258 staining assay

Hoechst 33258 staining was used to observe changes in the cell structure and apoptosis. In brief, SW1990 cells in logarithmic growth phase were cultured in six-well plates at a density of 2 × 10 ^5^ cells/well (*n* = 3) and exposed to different concentrations of XHP (0 μmol/L, 74.5 μg/mL, 92.7 μg/mL, 116.36 μg/mL, 145.45 μg/mL, and 181.82 μg/mL) for 24 h. The treated cells were then washed with PBS (−) twice and fixed at 4°C for 10 min with 1 mL of methanol per well. The cells were then incubated with Hoechst 33258 dye for 10 min at room temperature and washed 2–3 times with PBS (−). We then observed any change in cell structure by T 5 A/H fluorescence microscopy (Carl Zeiss, Germany). The resulting images were quantified using ImageJ V1.8.0.

### 2.7 Flow cytometry

SW1990 cells were seeded in six-well plates and incubated at 37°C in a 95% O_2_, 5% CO_2_ incubator for 24 h. To assess the effect of XHP on cell viability, five different concentrations of XHP and a control group were established and incubated for 24 h. The supernatant was collected from the six-well plates and the SW1990 cells were dissociated using EDTA-free trypsin. The collected supernatant was then added to the detached cells and centrifuged. The supernatant was discarded, and the cells were washed twice with pre-cooled PBS before being mixed and suspended for counting. Next, 10^5^ cells per tube were centrifuged at 1000 r/min for 5 min. The supernatant was discarded, and 195 µl of binding buffer was added and gently suspended. Subsequently, 5 µL of PI with 5 µI annexin V-FITC was added, the mixture was well agitated, and the final solution was incubated for 15 min at room temperature in the dark. Then, the solution was placed in an ice bath. Apoptosis was measured within 1 h using a B4 flow cytometer (ACEA NovoCyte, United States).

### 2.8 SW1990 PC cell xenograft model

SW1990 cells were cultured to a density of 1 × 10^7^ cells/mL at 37°C in a 95% O_2_, 5% CO_2_ incubator. Cells at ∼70–80% confluency were mixed with PBS (8121733, Gibco, United States). To build a tumor-bearing mouse model, 0.1 mL of cell suspension (approximately 2 × 10^6^ tumor cells) was subcutaneously injected near the right hind limb of nude mice. We measured the volume of the solid tumors after approximately 1 week using the following formula: $v=\frac{a\times b^{2}}{2}$ , where a is the length diameter and b is the short diameter. Once the tumor size was approximately 62.5 mm^3^, the mice were randomly divided into the following five groups (*n* = 6): model group (which received an equal volume of carboxymethylcellulose sodium, CMC-Na), 0.47 g/kg XHP group, 0.93 g/kg XHP group, 1.87 g/kg XHP group, and gemcitabine group (0.065 g/kg, as doses ≥0.0065 g/kg were lethal to mice). We report here on the use of XHP as a reference for the clinical therapeutic application at a specific dose based on a reported value of 1.233 g/kg, which was derived from a standard body weight of 60 kg and a standard body size factor of k = 12.33. We used a low dose of 0.47 g/kg and a high dose of 1.87 g/kg based on the XHP ratio (0.76 times that of XHW). Therefore, the medium dose was 0.93 g/kg. For the gemcitabine group, we considered 0.065 g/kg, as ≥0.0065 g/kg is lethal to mice. Subsequently, the XHP group received daily gavage administration, while the model group was administered CMC-Na once daily for 12 days. In the gemcitabine group, nude mice were intraperitoneally injected with gemcitabine at a dose of 0.065 g/kg once every 6 days, for a total of two doses. After completing the experiment, we measure the sizes of the solid tumors every 3 days based on the tumor tissue mass using the following formula: ( $Tumor\;inhibition\;rate\left(\%\right)=\left(\frac{W_{Model}-W_{XHP}}{\bar{W}}\right)\times 100\%$ ). To follow up the aforementioned solid mass measurement, the animals were anesthetized by urethane injection. The tumor tissue was fixed in 4% paraformaldehyde (142174, Biosharp, China) and embedded in paraffin.

### 2.9 Immunocytochemical analysis of the antiproliferative activity of XHP

To perform the immunocytochemical assay, we followed the standard method using cell nuclear antigen Ki67 (1:100). In brief, the collected samples fixed in formalin were treated with 3% H_2_O_2_ to block endogenous peroxidase activity and then incubated with 10% goat serum (1:9) before an overnight incubation at 4°C with anti-Ki67 antibodies. Next, the samples were incubated with secondary antibodies. Finally, staining was carried out using the DAB kit. The immunoreactivity was then determined using a blinded method in which we counted the total number of positive antigen cells in five high-power fields (40×) per section.

### 2.10 TUNEL apoptosis

To check for apoptosis signs, the cells in the collected samples were stained using the TUNEL (TdT-mediated dUTP nick end-labeling) apoptosis detection kit followed by image processing using a BA210 digital trinocular camera microscope (MOTIC, United States). The image observation was performed for each sample at low magnification of three representative areas (×400). The percentage of positive expression was calculated using the Image-Pro Plus 6.0 image analysis system (Media Cybernetics, United States).

### 2.11 Widely targeted metabolomics analysis

The metabolomics samples were prepared as described elsewhere. In brief, samples stored at −80°C were first thawed on ice. The thawed samples were homogenized using a grinder (30 HZ) for 20 s. Then, 400 μL solution (Methanol: Water = 7:3, V/V) containing internal standard was added to 20 mg of ground sample followed by vigorous agitation for 5 min. After incubating on ice for 15 min, the samples were centrifuged at 12,000 rpm for 10 min (4°C). Next, 300 μL of supernatant was collected and placed at −20°C for 30 min before centrifugation at 12,000 rpm for 3 min (4°C). Finally, 200 μL aliquots of supernatant were transferred for further LC-MS analysis.

T3 UPLC: Extracted samples were analyzed on an LC-ESI-MS/MS system (UPLC, ExionLC AD, https://sciex.com.cn/; MS, QTRAP® System, https://sciex.com/) using the following conditions: UPLC: column, Waters ACQUITY UPLC HSS T3 C18 (1.8 µm, 2.1 mm*100 mm); column temperature, 40°C; flow rate: 0.4 mL/min; injection volume, 2 μL; solvent system: water (0.1% formic acid), acetonitrile (0.1% formic acid); and gradient program: 95:5 V/V at 0 min, 10:90 V/V at 11.0 min, 10:90 V/V at 12.0 min, 95:5 V/V at 12.1 min, and 95:5 V/V at 14.0 min.

ESI-QTRAP-MS/MS: Both LIT and triple quadrupole (QQQ) scans were set up on a triple quadrupole-linear ion trap mass spectrometer system (QTRAP® LC-MS/MS System). The scanner was fixed with an ESI turbo ion-spray interface controlled by Analyst 1.6.3 software (Sciex). We set up operation functions based on the following optimized conditions: source temperature: 500°C; ion spray voltage (IS): 5500 V (positive), −4500 V (negative); ion source gas I (GSI), gas II (GSII), and curtain gas (CUR): 55, 60, and 25.0 psi, respectively. For instrument tuning and mass calibration, we used 10 and 100 μmol/L polypropylene glycol solutions in QQQ and LIT modes. The MRM transitions were monitored for each period at a specific set.

### 2.12 Analysis of metabolomics data

We performed qualitative evaluations of the detected substances using the MetWare database (MWDB) with reference to retention time (RT), ion-pair information, and secondary spectral data followed by multiple reaction monitoring (MRM) analysis utilizing triple quadrupole mass spectrometry. Quality control analysis and mass spectrometry data were processed using Analyst 1.6.3 software, while principal component analysis (PCA) was performed using the statistical function prcomp within the R programming language (www.r-project.org) followed by processing the data to UV (unit variance scaling). Heatmaps were generated using the ComplexHeatmap package in R software to perform hierarchical cluster analysis (HCA) of the metabolite accumulation patterns across the samples. To select differential metabolites (VIP≥1 and |log2FC|≥1.0), orthogonal least squares-discriminant analysis (OPLS-DA) was used to observe the classification of the two groups (Thévenot et al., 2015). The identified metabolites were then annotated using the KEGG compound database (http://www.kegg.jp/kegg/compound/) and pasted into the KEGG pathway database (http://www.kegg.jp/kegg/pathway.html). We then assessed the potential biological roles of the relevant differential metabolites using the MetaboAnalyst enrichment analysis database (http://www.metaboanalyst.ca/).

To build each metabolite and its optimized targeting, we used MetScape (http://metscape.ncibi.org/) as described by Gao et al. (2010) and Obi et al. (2016). To identify the interactions of proteins in their internal structures, we built those interactions in STRING (https://cn.string-db.org/) based on the aforementioned targets and identified key targets using Cytoscape’s MCODE plugin and UALCAN online database analysis (Chandrashekar et al., 2017; Feng et al., 2019; Chandrashekar et al., 2022).

### 2.13 Western blot

To perform Western blot analysis, each selected tumor was lysed in RIPA solution (G2002, Servicebio, China) with steel beads in a KZ-III-F high-speed cryogenic tissue grinder (Servicebio, China). We measured the protein amount using a BCA protein quantification kit. In brief, the lysed sample (100 µg) was loaded on 8%–12% polyacrylamide gels and transferred to PVDF membranes. TBST buffer diluted with 5% skimmed milk was then used to block the membranes for 1.5 h, followed by incubation with CYP3A4 and GAPDH antibodies for 8–12 h, respectively. The membranes were then incubated in biotinylated goat antibody IgG for 1 h.

### 2.14 RT-PCR

Reverse transcription PCR (RT-PCR) was performed using RNA extracted from the cells using TRIzol reagent. The mRNA levels were measured in a reaction using SYBR Green PCR mix, as previously described. The primers for CYP3A4 (tta​tgc​tct​tca​cca​tga​ccc​aca​g and caa​tgc​tgc​cct​tgt​tct​ctt​tgc) and β-actin (cta​cct​cat​gaa​gat​cct​gac​c and cac​agc​ttc​tct​ttg​atg​tca​c) were designed based on the NCBI database. The ΔΔCT method was used to determine the relative gene expression levels with β-actin levels as a reference, as previously described.

### 2.15 Statistical analysis

One-way ANOVA was performed using GraphPad Prism v6.0 and SPSS 23.0. The data were expressed as means ± standard deviation (±SD). *p* < 0.05 was considered statistically significant.

## 3 Results

### 3.1 UPLC/Q-TOF-MS determination of the main components of XHP

**FIGURE 1:**
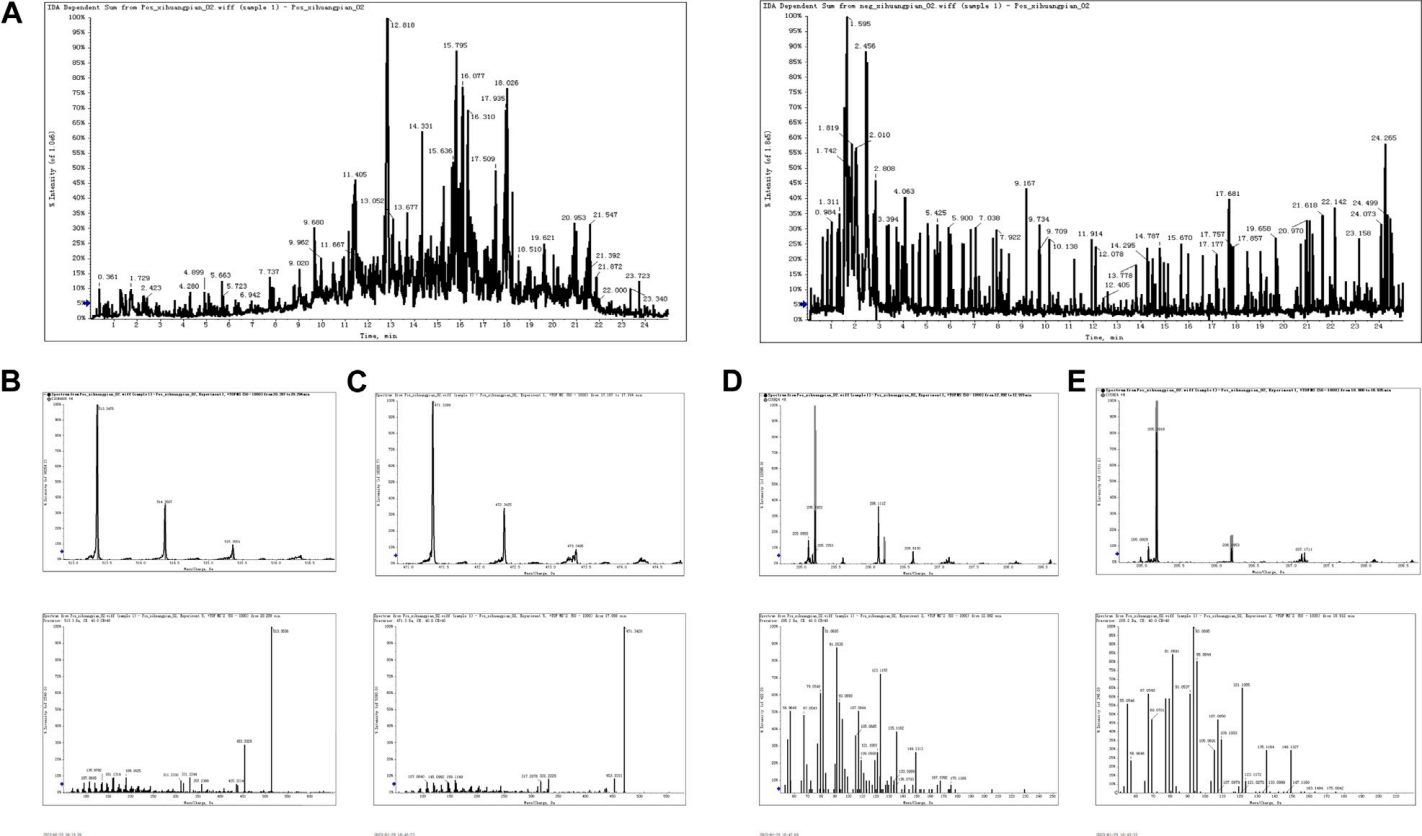
UPLC/Q-TOF-MS for the determination of the main components of XHP **(A)**. TIC in positive and negative ion modes of XHP. **(B)** Primary and secondary fragment ion plots of 11-carbonyl-β-acetyl boswellic acid (AKBA). **(C)** Primary and secondary fragment ion plots of 11-carbonyl-β-boswellic acid (KBA). **(D)** Primary and secondary fragment ion plots of 4-methylene-2,8,8-trimethyl-2-vinyl-bicyclo [5.2.0]nonane. **(E)** Primary and secondary fragment ion plots of (1S-endo)-2-methyl-3-methylene-2-(4-methyl-3--3-pentenyl)-bicyclo [2.2.1heptane.

As seen in Figure 1A, total chromatograms for both positive and negative ion modes were obtained according to the optimized condition of the spectrometric instrument. Analysis of the XHP components was based on all collected samples for the primary and secondary fragment ions for the optimized experimental condition. The main components of XHP identified by UPLC/Q-TOF-MS, were 11-carbonyl-β-acetyl boswellic acid (AKBA), 11-carbonyl-β-boswellic acid (KBA), 4-methylene-2,8,8 -trimethyl-2-vinyl-bicyclo [5.2.0]nonane, and [(1S-endo)-2-methyl-3-methylene-2-(4-methyl-3- pentenyl)-bicyclo 2.2.1] heptane (Table 1; Figures 1B–E).

**TABLE 1:** UPLC/Q-TOF-MS data analysis.

Name	Formula	Mass (Da)	Found at mass (Da)	tR (min)
AKBA	C_32_H_48_O_5_	513.3575	513.3485	20.28
KBA	C_30_H_46_O_4_	471.3469	471.3389	17.10
4-Methylene-2,8,8-trimethyl-2-vinyl-bicyclo [5.2.0]nonane	C_15_H_24_	205.1951	205.1922	12.88
(1S-endo)-2-Methyl-3-methylene-2-(4-methyl-3--3-pentenyl)-bicyclo [2.2.1heptane	C_15_H_24_	205.1951	205.1922	18.92

### 3.2 XHP inhibits SW1990 PC cell growth *in vitro*

**FIGURE 2:**
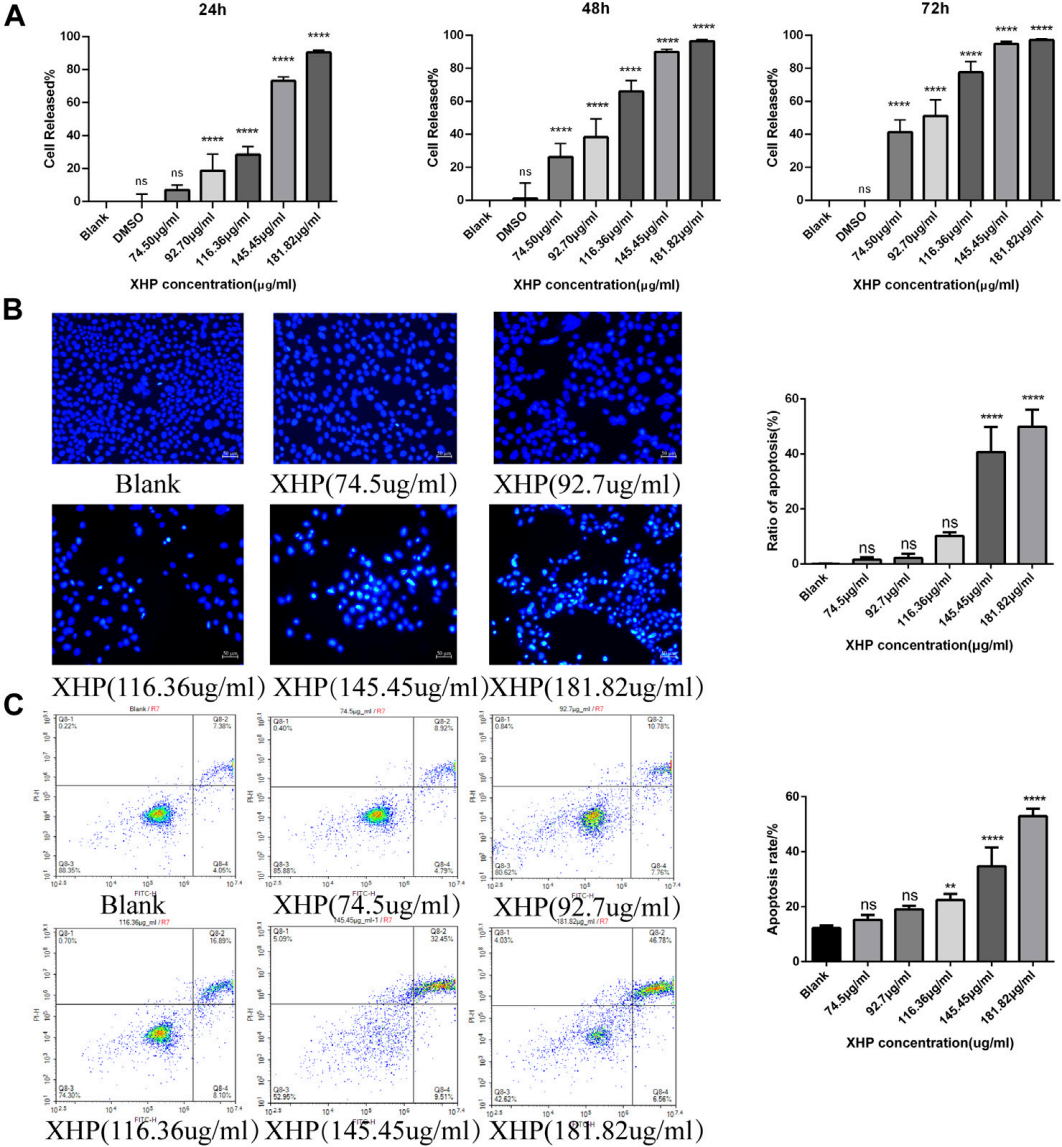
Effect of XHP on PC tumor growth *in vitro*. **(A)** Concentration and time-dependent inhibitory effect of XHP (92.7 μg/mL) on SW1990 cells after 24 h of treatment. **(B)** Hoechst 33258 staining assays to evaluate PC cell clonality after 24 h of XHP treatment. Scale bar, 50 µm. **(C)** Induction of SW1990 cell apoptosis after XHP treatment for 24 h. The apoptotic processes were evaluated by flow cytometry. **p < 0.01 and ****p < 0.0001.

To assess the significant anti-tumor effects of XHP on PC, we first cultured SW1990 PC cells in the presence of various XHP concentrations (74.5 μg/mL, 92.7 μg/mL, 116.36 μg/mL, 145.45 μg/mL, and 181.82 μg/mL). Untreated cells in DMSO were used as the control group. At the end of the experiment, we performed CCK-8 assays at different times (24, 48, and 72 h). As shown in Figure 2A XHP significantly inhibited PC cells in dose- and time-dependent manners. The inhibition rates are summarized in Table 2. DMSO did not significantly impact the results (*p* > 0.05). This finding demonstrates the anti-proliferative effects of XHP on PC cells.

**TABLE 2:** Inhibition rate of SW1990 pancreatic cancer cells by Xihuang tablets (±SD, *n* = 6).

Group	Concentration (µg/mL)	24 h (%)	48 h (%)	72 h (%)
Blank	0.00	0.00	0.00	0.00
DMSO	0.4%	0.067 ± 3.697	1.119 ± 7.624	−1.262 ± 3.073
XHP	74.50	7.014 ± 2.679	26.308 ± 7.308****	41.305 ± 7.385****
	92.70	18.698 ± 8.638****	38.330 ± 10.185****	51.236 ± 9.727****
	116.36	28.449 ± 4.610****	66.041 ± 5.981****	77.607 ± 6.457****
	145.45	73.180 ± 2.265****	90.036 ± 1.438****	94.874 ± 1.248****
	181.82	90.431 ± 0.924****	96.571 ± 0.480****	97.205 ± 0.443****

Compared to blank, each group ****p < 0.0001.

### 3.3 XHP promotes apoptosis in SW1990 PC cells *in vitro*

Figure 2B shows that the application of XHP had some pro-apoptotic effects on SW1990 human PC cells. With increasing concentrations of XHP (0 μg/mL, 74.5 μg/mL, 92.7 μg/mL, 116.36 μg/mL, 145.45 μg/mL, and 181.82 μg/mL), the apoptosis rate increased from 11.43% to 13.71%, 18.54%, 18.54%, 24.99%, 41.96%, and 53.34%, respectively (Figure 2C). Our data suggest that the percentage of SW1990 PC cells undergoing early apoptotic (annexin V + -PI-) and late apoptotic (annexin V + -PI+) stages increased in a dose-dependent manner with increasing XHP concentration. Notably, we observed a significant difference at concentrations >116.36 μg/mL (*p* < 0.01). Taken together, our results prove the pro-apoptotic properties of XHP in PC cells.

### 3.4 XHP inhibits the growth of PC xenograft models *in vivo*

**FIGURE 3:**
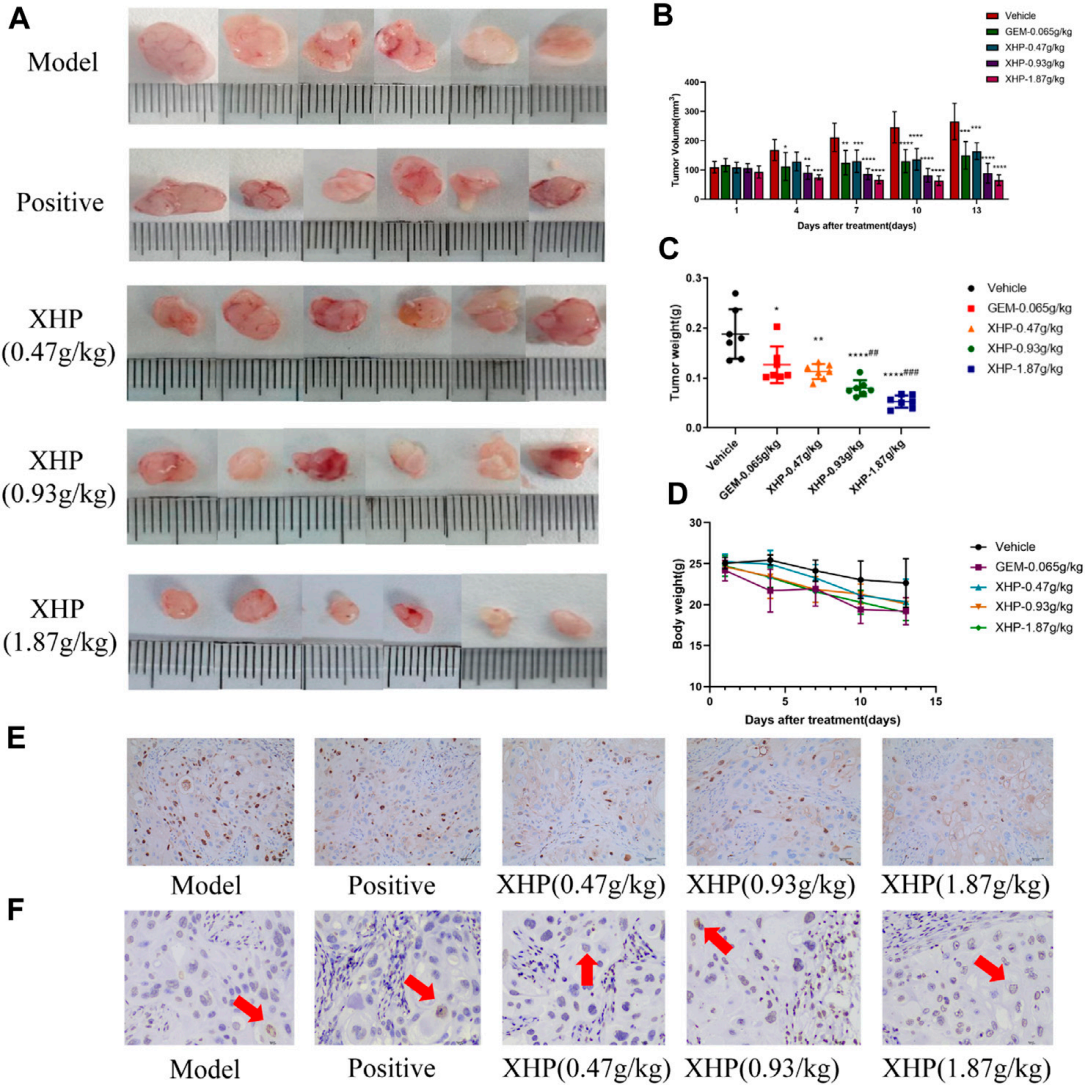
Effect of XHP on PC tumor growth *in vivo*. **(A)** Tumor dissection in an animal model of PC established by injecting an SW1990 cell suspension into BALB/c nude mice. **(B)** Tumor volume was recorded every 3 days during tumor growth. **(C)** Tumor weight was recorded after sacrifice. **(D)** Body weight of mice during tumor growth was recorded every 3 days. **(E)** Hematoxylin-stained nuclei (blue). DAB (brownish yellow) shows positive expression. Scale bar: 40 µm, magnification: ×20. **(F)** Positive expression: apoptotic cell nuclei light yellow or brownish yellow color, negative expression (normal cell nuclei): blue or light blue with white background. Scale bar: 10 µm, magnification: ×400. Compared with the model group, each group *p < 0.05, **p < 0.01, ****p < 0.0001; compared with the positive group, each XHP group ##*p* < 0.01, ###*p* < 0.001.

To further analyze the anti-PC effects of XHP, we subcutaneously injected SW1990 cells near the right hind limb of mice. Tumor volume and weight were measured every 3 days after treatment with different concentrations of XHP. The mice were euthanized after 12 days. Figures 3A–C show that XHP significantly reduced the tumor volume compared to the model group (**p < 0.01, ****p < 0.0001). Moreover, the tumor volume was lower at medium and high doses of XHP compared to those in the positive control group administered intraperitoneal gemcitabine hydrochloride (###*p* < 0.01, ###*p* < 0.001). No significant differences in the body weight of mice were observed between the positive control and XHP groups compared to the model group (Figure 3D). Therefore, our data proved that XHP inhibited the growth of PC in the mouse model. Additionally, the immunohistochemical assay demonstrated that XHP significantly reduced the expression level of Ki67 in a dose-dependent manner in the tumor tissues of PC mice (Table 3; Figure 3E) and showed stronger anti-proliferative effects compared to the positive control group.

**TABLE 3:** umbers of Ki67-positive positive control cells (±SD).

Group	Sample	Positive nuclei ( $\bar{x}$ ± SD)
Model	3	89.44 ± 4.99
Positive control (0.065 g/kg)	3	55.89 ± 10.31^***^
XHP (0.47 g/kg)	3	42.56 ± 4.35^****^
XHP (0.93 g/kg)	3	31.33 ± 2.60^****##^
XHP (1.87 g/kg)	3	14.89 ± 5.36^****###^

All groups compared to the model group, ***p < 0.001, ****p < 0.0001; XHP all dose groups compared to the positive group, ##*p* < 0.05, ##*p* < 0.001.

### 3.5 XHP has pro-apoptotic effects on PC cells *in vivo*

TUNEL analysis was applied to evaluate the pro-apoptotic effect of XHP on PC tumors (Table 4; Figure 3F). We observed that XHP significantly affected the apoptotic rate in tumor tissues compared to the model group (*p* < 0.0001). We also observed a dose-dependent pro-apoptotic effect in the XHP group. The significant value in the XHP group (at doses of 0.93 g/kg and 1.87 g/kg) showed higher apoptosis rates in tumor tissues (*p* < 0.001). A significantly lower apoptosis rate was observed in the 0.47 g/kg XHP group (*p* < 0.05).

**TABLE 4:** Percentages of apoptotic cells in the tumor tissues of nude mice (%). Statistical results (±SD).

Group	Sample	Positive nuclei ( $\bar{x}$ ± SD)
Model	3	2.35 ± 0.44
Positive control (0.065 g/kg)	3	8.74 ± 0.44^****^
XHP (0.47 g/kg)	3	6.66 ± 0.63^****#^
XHP (0.93 g/kg)	3	12.78 ± 0.05^****###^
XHP (1.87 g/kg)	3	19.28 ± 1.18^****####^

All groups compared to the model group, ****p < 0.0001; XHP: all dose groups compared to the positive group, #*p* < 0.05, ###*p* < 0.001, ####*p* < 0.0001.

### 3.6 Widely targeted metabolomics analysis identifies differential metabolites and metabolic pathways

**FIGURE 4:**
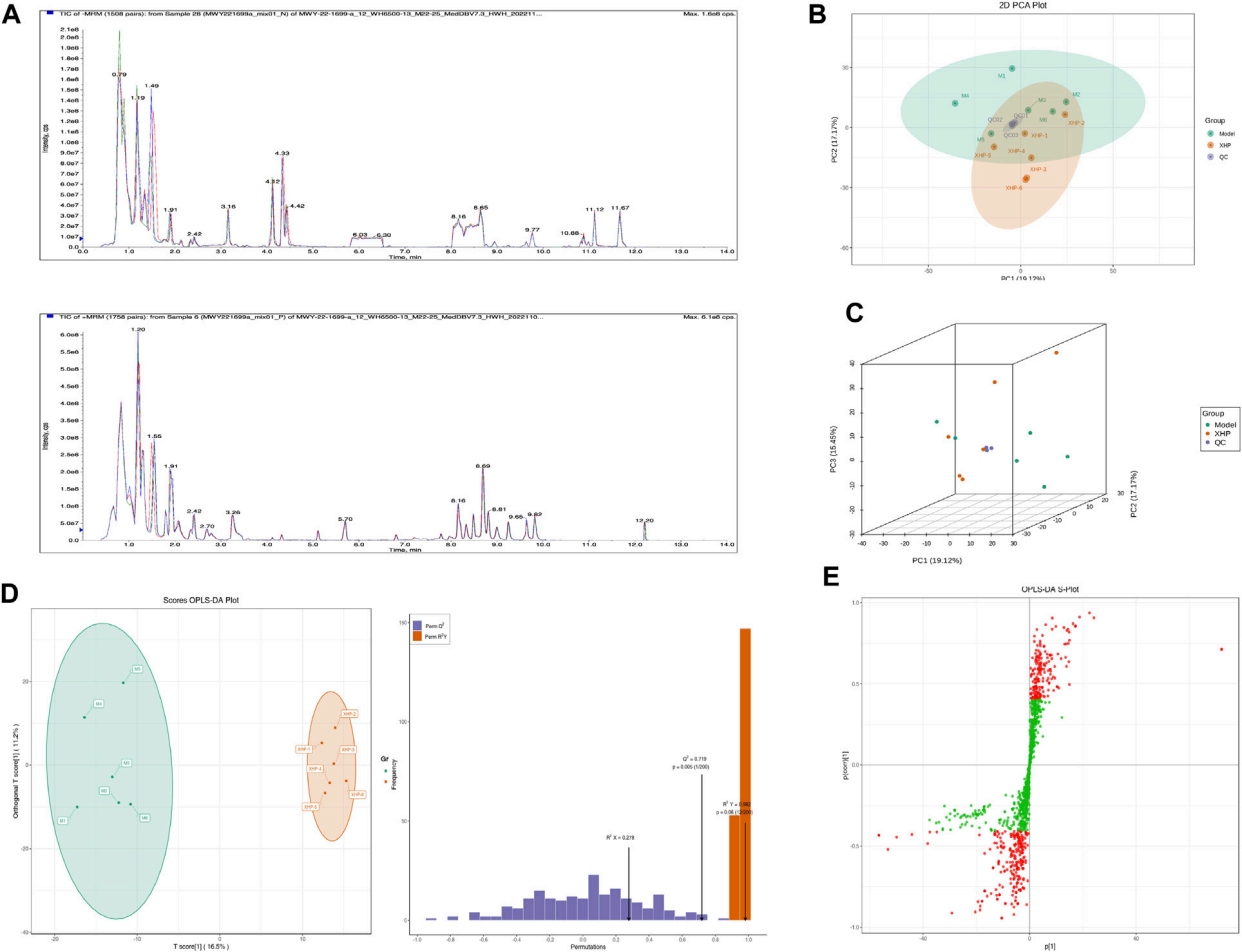
Plots for multivariate statistical analysis (1). **(A)** Superimposed plot of the total ion flow diagram (TIC plot) for the mass spectrometric detection of QC samples. N, negative ion mode; P, positive ion mode. **(B)** PCA-2D plot. PC1–PC3, first, second, and third principal components, respectively. Percentage, interpretation rate of the principal component to the data set. **(C)** PCA-3D plot: PC1–PC3, first, second, and third principal components, respectively. **(D)** OPLS-DA score plot. Horizontal coordinate, predicted principal component; horizontal direction, gap between groups; vertical coordinate, orthogonal principal component; vertical direction, gap within groups; percentage, explanation rate of the component to the data set (R2X (cum) = 0.278; R2Y (cum) = 0.982; Q2 (cum) = 0.719; pre = 1; ort = 1; *p* = 0.005). **(E)** S-plot of OPLS-DA. Horizontal coordinate, covariance between the principal component and the metabolite; vertical coordinate, correlation coefficient between the principal component and the metabolite. The closer the metabolite is to the upper right and lower left corner, the more significant the difference. Red dots, VIP ≥ 1; green dots, VIP ≤ 1. Each dot in the plot indicates a sample, with samples from the same group represented using the same color.

**FIGURE 5:**
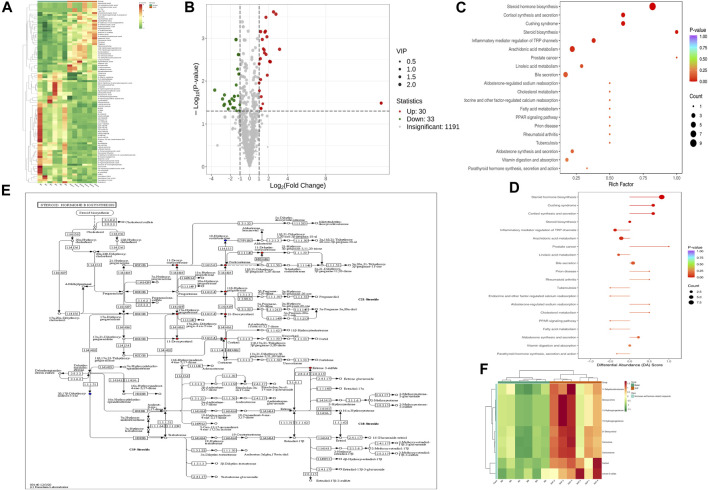
Plots for multivariate statistical analysis (2). **(A)** HCA diagram: horizontal, sample name; vertical, differential metabolite information; red, high content; green, low content; darker color, higher content; clustering line on the left side of the diagram, metabolite clustering line. **(B)** Volcano plot. Each point in the volcano plot represents a metabolite. Green, downregulated; red, upregulated; gray, metabolites detected but not significantly different; horizontal coordinate, |log_2_FC| of the relative content difference of a metabolite in two groups of samples. The larger the absolute value of the horizontal coordinate, the larger the relative content difference between the two groups of samples. Under the VIP + FC + *p*-value triple screening condition: vertical coordinate, level of significance of the difference (-logP-value); dot size, VIP value. **(C)** KEGG enrichment analysis of the top 20 differential metabolites. Horizontal coordinate, rich factor for each pathway, with larger values indicating greater enrichment; vertical coordinate, pathway name (sorted by *p*-value), dot color, *p*-value magnitude, with more red indicating more significant enrichment. **(D)** Analysis of the overall changes in the KEGG pathways of the top 20 differential metabolites. Vertical coordinate, differential pathway name (sorted by *p*-value); horizontal coordinate, differential abundance (DA) score; line segment length, absolute value of the DA score; dot size at the end of the line segment, number of differential metabolites in that pathway. Larger dots indicate a higher number of metabolites. The colors of the line, and the dots reflect the magnitude of the *p*-value, with closer to red indicating a smaller *p*-value and closer to purple indicating a larger *p*-value. **(E)** Annotated graph of metabolite function. Red, metabolite content significantly upregulated in the experimental group; blue, metabolite detected but not significantly changed; green, metabolite content significantly downregulated in the experimental group. **(F)** Cluster analysis plot of differential metabolites of the KEGG pathway. Horizontal coordinates, sample names; vertical coordinates, differential metabolites; red, high levels; green, low levels.

To assess the effects of XHP on the metabolic mechanism of the PC model, we identified and analyzed differential metabolites in mouse tumor tissue. We then performed TIC of mass spectrometry for different QC samples of the model and XHP groups. Our data showed higher degrees of similarity in the curves for the total ion current of the metabolite assay, indicating consistent retention times and peak intensities. These results were in good accordance with the MS results when the same sample was detected at different times (Figure 4A). PCA analysis (Figures 4B, C) and OPLS-DA (Figures 4D, E) showed clear metabolome separation between the model and XHP groups, Heatmaps were then generated using the ComplexHeatmap package in R after UV (unit variance scaling) processing, and hierarchical cluster analysis (HCA) was applied to the accumulation patterns of 93 metabolites across different samples. Of these metabolites, 36 were upregulated and 57 were downregulated (Figure 5A). A volcano scatter plot (Figure 5B) was generated to display the results of screening 63 differential metabolites based on the triple screening principle of VIP≥1, FC ≥ 2 or FC ≤ 0.5, and *p* < 0.05. Each metabolite was represented by a dot on the plot, with the degree of variation indicated by different colors. Each row in the plot represented one sample, and each column represented one metabolite. The top 20 differential metabolites are shown in Table 5. The differential metabolite results were subjected to KEGG pathway enrichment analysis (Figures 5C, D), which identified five significantly enriched metabolic pathways, including steroid hormone biosynthesis, cortisol synthesis and secretion, Cushing’s syndrome steroid biosynthesis, steroid biosynthesis, and inflammatory mediator regulation of TRP channels. The top ten metabolites were annotated using the KEGG database (Kanehisa and Goto, 2000), which showed that the major metabolic pathway was steroid hormone biosynthesis (Figure 5E). To further analyze the differential metabolites identified based on the screening criteria, the significantly enriched KEGG metabolic pathways were selected and all differential metabolites in these pathways were clustered (Figure 5F). The clustered metabolites included Ile-Tyr, Leu-Tyr, tripterin, cortisol, urobilin, 11β-hydroxyprogesterone, 17α-hydroxyprogesterone, and desoxycortone, among others.

**TABLE 5:** Top 20 ranked differential metabolites.

Index	Formula	Compound	Class I	VIP	*p*-value	FC	Type
MEDN2000	C_15_H_22_N_2_O_4_	Ile-Tyr	Amino acid and its metabolites	1.75E+00	3.26E-02	1.44E+04	Up
MEDN1999	C_15_H_22_N_2_O_4_	Leu-Tyr	Amino acid and its metabolites	1.75E+00	3.26E-02	1.44E+04	Up
MEDP2286	C_29_H_38_O_4_	Tripterin	Benzene and substituted derivatives	2.23E+00	1.81E-03	8.88E+00	Up
MEDP0889	C_21_H_30_O_5_	Cortisol	Hormones and hormone-related compounds	2.31E+00	2.74E-04	6.82E+00	Up
MEDP1255	C_33_H_42_N_4_O_6_	Urobilin	Tryptamines, cholines, and pigments	2.24E+00	2.42E-04	5.71E+00	Up
MEDP1709	C_21_H_30_O_3_	11β-Hydroxyprogesterone	Hormones and hormone-related compounds	2.11E+00	3.54E-03	4.51E+00	Up
MEDP1636	C_21_H_30_O_3_	17α-Hydroxyprogesterone	Hormones and hormone-related compounds	2.11E+00	3.54E-03	4.51E+00	Up
MEDP1635	C_21_H_30_O_3_	Desoxycortone	Hormones and hormone-related compounds	2.11E+00	3.54E-03	4.51E+00	Up
MEDN0105	C_26_H_45_NO_7_S	Taurocholic acid	Bile acids	2.14E+00	3.48E-03	4.32E+00	Up
MEDP0315	C_9_H_9_NO_3_	Hippuric acid	Organic acid and its derivatives	1.89E+00	7.28E-03	3.63E+00	Up
MEDN1493	C_4_H_8_O_3_	3-Hydroxybutanoic acid	Organic acid and its derivatives	1.48E+00	3.38E-02	2.44E-01	Down
MEDN0749	C_22_H_32_O_4_	1-Mar	FA	1.91E+00	2.97E-02	2.32E-01	Down
MEDN0790	C_22_H_32_O_4_	PDX	FA	1.91E+00	2.97E-02	2.32E-01	Down
MEDN0802	C_22_H_32_O_4_	RvD5	FA	1.91E+00	2.97E-02	2.32E-01	Down
MEDN0771	C_20_H_30_O_3_	15-oxoETE	FA	1.99E+00	4.47E-02	2.13E-01	Down
MEDN1444	C_22_H_32_O_3_	16-HDoHE	FA	2.14E+00	3.48E-02	1.62E-01	Down
MEDN0375	C_18_H_30_O_3_	13-HOTrE	FA	2.07E+00	2.10E-02	1.62E-01	Down
MEDN1442	C_22_H_32_O_3_	8-HDoHE	FA	2.20E+00	3.69E-02	1.55E-01	Down
MEDN0769	C_22_H_32_O_3_	14(S)-HDHA	FA	2.23E+00	2.61E-02	1.40E-01	Down
MEDN0754	C_22_H_32_O_3_	(±)17-HDHA	FA	2.25E+00	1.62E-02	7.94E-02	Down

### 3.7 CYP3A4 is a key target for the anti-PC effects of XHP

**FIGURE 6:**
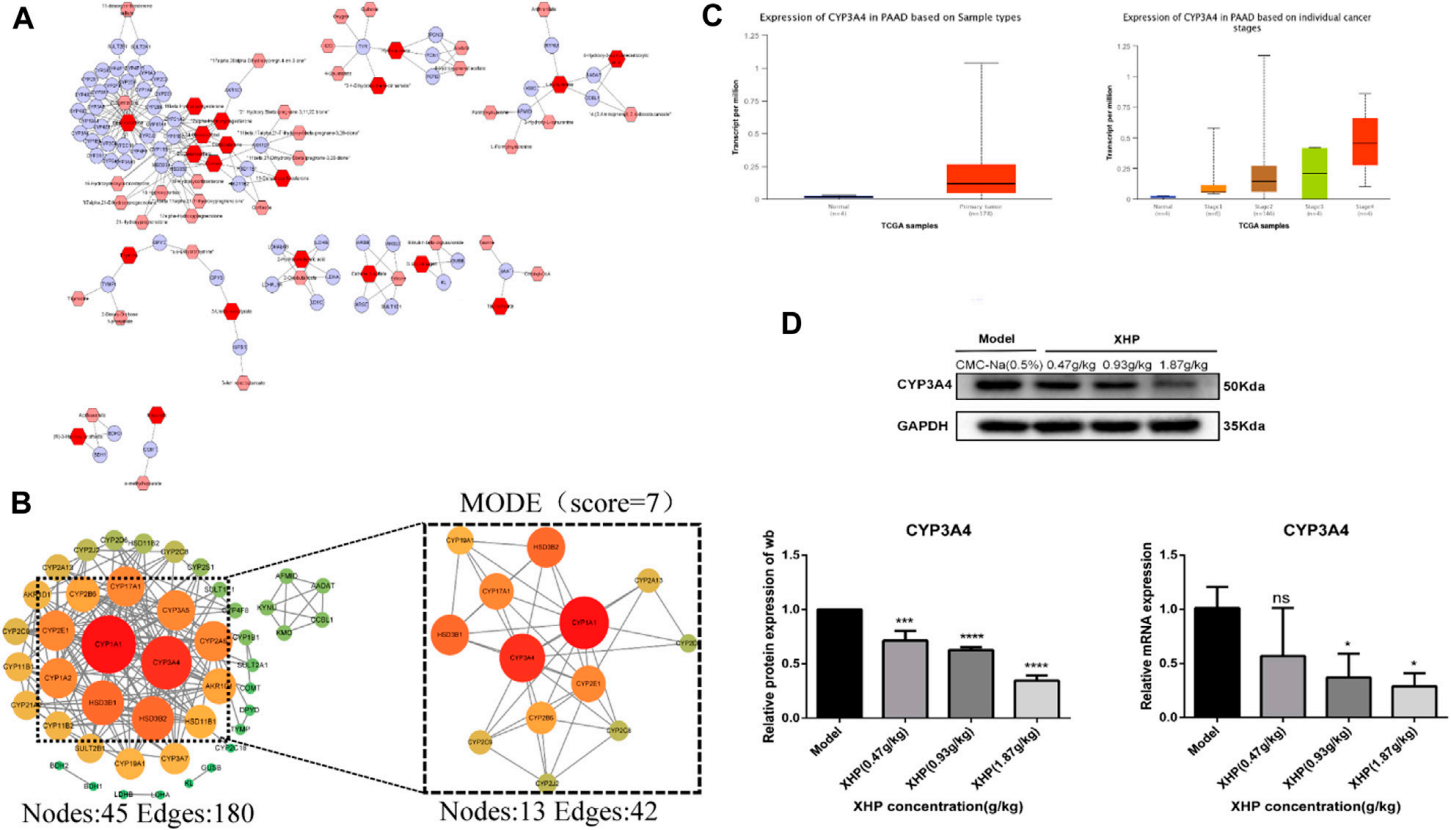
CYP3A4 may be a candidate key target for XHP in the treatment of PC. **(A)** Differential metabolite composition–target network diagram. Nodes, relevant metabolites, and targets regulated by important metabolites; edges, biochemical reactions. **(B)** PPI networks of the aforementioned metabolite regulatory targets and screening of candidates by MCODE for the highest scoring key targets. Darker colors and larger circles, larger degree values. **(C)** CYP3A4 as a potential key target in PC screened by the UALCAN online database (*p* < 0.05). **(D)** Expression levels of CYP3A4 in XHP-treated and control pancreatic cancer (PC) cells by both Western blot (WB) and reverse transcription polymerase chain reaction (RT-PCR). *p < 0.05, ***p < 0.001, ****p < 0.0001.

Differential metabolites were selected based on the following criteria: VIP value ≥ 1, fold change ≥2 or ≤0.5, and *p* ≤ 0.05. The known KEGG IDs of the selected metabolites were obtained using MetScape (Cytoscape plug-in). The ID information obtained in the previous step was then imported into the system to construct a metabolite composition–target network (Figure 6A). A total of 64 relevant targets were identified. A protein–protein interaction (PPI) network was constructed using the STRING platform. Targets outside of the network were excluded, and the remaining targets were imported into Cytoscape and arranged based on their degree values (Figure 6B). To further refine the target selection, the MODE plug-in was used to obtain 13 significant targets with the highest scores (score = 7) from the reciprocal map of targets. Additionally, KEGG enrichment analysis revealed that these targets were significantly associated with the steroid hormone biosynthesis metabolic pathway. The 13 targets were subsequently analyzed in the UALCAN online database to identify key targets, among which CYP3A4 was identified as a crucial target (Figure 6C). Targets with *p* > 0.05 were considered non-significant and were excluded from further analysis. Western blot (WB) experiments indicated that XHP treatment resulted in the downregulation of CYP3A4 protein expression (Figure 6D), while the RT-PCR experiments also showed that XHP downregulated the mRNA expression of CYP3A4 *in vivo*. These results suggested that CYP3A4 may be a useful key target for XHP in the treatment of PC.

## 4 Discussion

In the concept of biology, metabolites can be formed according to the organism phenotype, in which cellular interconnection is key for understanding the mechanisms to provide a more comprehensive view of metabolite changes (Kuang et al., 2021). Among the pathways underlying metabolomics, targeted tumor therapy is considered one successful therapy (Dang et al., 2010; Johnson et al., 2016). Therefore, drug targeting and metabolic pathways for cancer treatment are the core of therapeutic applications. Traditional Chinese medicine indicates that the interaction of dampness, heat, and toxicity may cause PC formation. In this context, XHP is primarily intended to soften the knots and disperse the canker sores consistent with the pathogenesis of PC. While previous studies demonstrated that the combination of XHW and gemcitabine can alleviate clinical symptoms in patients with PC, the direct anti-PC effects of XHW have not yet been reported. To our knowledge, this study is the first to demonstrate the significant efficacy of XHP in the treatment of PC. Moreover, we identified new targets of PC using metabolomics by screening widely targeted metabolites. The results of this analysis revealed that XHP not only possesses anti-PC properties but also has potential inhibitory effects on CYP3A4.

Our results showed that the administration of XHP had anti-PC cancer effects. XHP directly inhibited SW1990 PC cell activity with anti-proliferative and pro-apoptotic effects. Additionally, the animal model showed that XHP significantly inhibited tumor growth. XHP is composed of four Chinese herbal medicines; namely, Ruxiang, Moyao, artificial Niuhuang, and artificial Shexiang. Our quality analysis showed that XHP mainly consists of 11-carbonyl-β-acetyl boswellic acid (AKBA), 11-carbonyl-β-boswellic acid (KBA), 4-methylene-2,8,8 -trimethyl-2-vinyl-bicyclo [5.2.0]nonane, and (1S-endo)-2-methyl-3-methylene-2-(4-methyl-3- pentenyl)-bicyclo [2.2.1heptane. In addition, AKBA and KBA have anti-inflammatory effects by inhibiting the production of pro-inflammatory cytokines, such as interleukin-1β (IL-1β), interleukin-6 (IL-6), and tumor necrosis factor-α (TNF-α), and suppressing the activation of nuclear factor kappa-light-chain-enhancer of activated B cells (NF-κB) (Siddiqui, 2022). KBA induced apoptosis and cell cycle arrest at the G2-M phase in non-small cell lung cancer H446 cells (Agrawal et al., 2011). AKBA significantly suppressed pro-inflammatory factors in tumor tissue and inhibited biomarkers of tumor survival, proliferation, aggressiveness, and angiogenesis, resulting in reduced growth and metastasis of human colorectal cancer *in vivo* (Huang et al., 2018). In addition, AKBA hindered gastric cancer cell proliferation and migration and promoted cell apoptosis through the PTEN/Akt/COX-2 signaling pathway (Yadav et al., 2012). Therefore, XHP exhibits significant anti-tumor effects.

Additionally, we found that the oral administration of XHP in the PC mouse model greatly impacted the metabolic profiles. Cellular metabolism represents a primary characteristic by orchestrating aberrant metabolic reprogramming to fulfill the increased energy requirements for sustained proliferation (Sun et al., 2020). Cancer cells can evade apoptosis through aberrant metabolic regulation (Kumar et al., 2022). Our findings showed that XHP induced alterations in the concentrations of multiple metabolites in PC mice, suggesting that XHP may modulate metabolism. Within the XHP-treated cohort, we observed notable modifications in the levels of metabolites such as fatty acyls and hormones relative to those in the model group. Genetic variation in pertinent genes can perturb steroid hormone biosynthetic pathways and their receptors, thereby modifying an individual’s susceptibility to gastric cancer (Pan et al., 2022). Based on this concept, the modulation of steroid hormone biosynthesis may be a promising anti-tumor pathway; however, further studies are needed.

We used MetScape to construct a metabolite component-target network based on the differential metabolites identified through the metabolomics analysis. Our results identified CYP3A4 as a potential key target for XHP treatment of PC, which is implicated in the biosynthesis of steroid hormones. The cytochrome P450 family (CYP) is a group of proteins that require heme as a cofactor. While the liver is the primary location of CYP-mediated drug metabolism, CYP enzymes are also expressed at varying levels in extrahepatic tissues, particularly in the small intestine, as well as the kidneys, lungs, and brain. Moreover, CYP is expressed in a range of tumor tissues (Cho et al., 2012). CYP plays a crucial role in the metabolism of diverse exogenous substances that can influence tumorigenesis by activating or deactivating carcinogens, and are also closely associated with chemical carcinogenesis (Van Eijk et al., 2019). Cancer therapy depends on the activity of the cytochrome P450 enzyme family, primarily carried out by CYP3A4 and CYP3A5 (Šemeláková et al., 2021). CYP3A4 is involved in the metabolism of over 50% of clinically active drugs; thus, its overexpression can lead to reduced efficacy and the development of chemotherapeutic drug resistance, posing a major challenge for patients with cancer (Lehmann et al., 1998; Sevrioukova and Poulos., 2013). Clinically, while CYP3A inhibition can present challenges such as inadvertent elevation of substrate drug exposure, resulting in toxicity, it may also offer benefits. For instance, inhibition can be advantageous because a substantial number of drugs are rapidly metabolized by CYP3A, leading to inadequate therapeutic plasma levels (Loos et al., 2022). CYP3A4 is primarily found in the liver and intestinal tissues. However, the expression levels of CYP3A4 in the intestine are not correlated with those in the liver, indicating the independent expression of CYP3A4 in different tissues (Ohno et al., 2007). Although CYP3A4 is not exclusively associated with PC, according to the UALCAN database, CYP3A4 is highly expressed in PC tissues and exhibits low expression in normal pancreatic tissues. Our results further revealed that XHP can suppress CYP3A4 expression, implying that CYP3A4 could be a therapeutic useful target for PC treatment. However, additional investigations are required to validate the specific mechanism of action of XHP in regulating CYP3A4. Thus, XHP could serve not as only an effective anti-PC therapeutic agent but also as a CYP3A4 inhibitor, similar to drugs such as ritonavir and cobicistat (Sevrioukova and Poulos, 2010; Sherman et al., 2015). Additionally, XHP may play a critical role in inhibiting resistance to chemotherapy drugs; however, further validation is necessary to confirm this hypothesis.

Our further validation of specific marker protein levels of XHP pro-PC apoptosis provides a more comprehensive understanding of the therapeutic effects of XHP. Further exploration of the mechanism of CYP3A4 inhibition by XHP may help to identify specific targets and pathways for future drug development. In addition, investigating which component of XHP is responsible for down-regulating CYP3A4 can inform the development of more targeted and effective treatments for PC. Also, combining XHP with PC chemotherapeutic agents and testing its ability to inhibit chemotherapeutic drug resistance by suppressing CYP3A4 expression is an avenue for future research that could provide insights into the potential clinical use of XHP as an adjunct therapy for the treatment of PC.

## 5 Conclusion

Our results demonstrated that XHP hindered the proliferation of PC cells and stimulated apoptosis both *in vivo* and *in vitro*. In addition, the metabolomics analysis showed that XHP elicited significant anti-PC effects by modulating the biosynthetic metabolic pathway of steroid hormones. Furthermore, XHP is not only an effective therapeutic agent against PC but may also function as a CYP3A4 inhibitor. In conclusion, our investigation utilized the widely targeted metabolomics method to screen for pivotal therapeutic targets through differential metabolites, thereby providing novel insights for exploring the role of herbal medicine and its compounding in disease treatment.

## Data Availability

The original contributions presented in the study are included in the article/Supplementary Material. Further inquiries can be directed to the corresponding authors.
